# Nonlinear relationship between oxidative balance score and hyperuricemia: analyses of NHANES 2007–2018

**DOI:** 10.1186/s12937-024-00953-1

**Published:** 2024-05-04

**Authors:** Fengmin Liu, Fangqin You, Lihang Yang, Xiaojuan Du, Cheng Li, Geng Chen, Diya Xie

**Affiliations:** 1 Department of Endocrinology, Fuzhou First General Hospital Affiliated with Fujian Medical University, Fuzhou, Fujian 350009 China; 2 Department of General Surgery, Fuzhou First General Hospital Affiliated with Fujian Medical University, Fuzhou, Fujian 350009 China; 3 Nursing Department, Fuzhou First General Hospital Affiliated with Fujian Medical University, Fuzhou, Fujian 350009 China

**Keywords:** NHANES, Oxidative balance score, Hyperuricemia, RCS, Oxidative stress

## Abstract

**Background:**

Limited data regarding the correlation between oxidative balance score (OBS) and hyperuricemia highlights the necessity for thorough investigations. This study aims to examine the link between OBS, which incorporates dietary and lifestyle factors, and the occurrence of hyperuricemia.

**Methods:**

We conducted a cross-sectional study involving 13,636 participants from the 2007–2018 National Health and Nutrition Examination Survey (NHANES). The oxidative balance score (OBS) was determined based on four lifestyle factors and sixteen dietary nutrients. We assessed the levels of serum uric acid (SUA) and the occurrence of hyperuricemia as outcomes. Weighted logistic regression and linear models were used for statistical analysis, using Restricted Cubic Splines (RCS) to examine potential nonlinear associations. Subgroup analysis and sensitivity assessments were performed to identify any variations and ensure the robustness of the findings.

**Results:**

Higher OBS was consistently correlated with decreased SUA levels and a reduced prevalence of hyperuricemia. RCS highlighted a significant negative nonlinear association, particularly in females. Subgroup analysis revealed gender-based differences and interactive correlation, providing additional insights regarding OBS and hyperuricemia relationship.

**Conclusion:**

This study underscores a robust negative correlation between OBS and SUA levels as well as the incidence of hyperuricemia, emphasizing the importance of dietary and lifestyle factors. Incorporating RCS, subgroup analysis, and sensitivity assessments enhances the depth of our findings, providing valuable insights for further research.

**Supplementary Information:**

The online version contains supplementary material available at 10.1186/s12937-024-00953-1.

## Introduction

Uric acid is the final byproduct of purine metabolism in humans. The kidneys eliminate around two-thirds of uric acid as free sodium urate in urine, while the remaining is either excreted through the intestines or broken down by intestinal bacteria [1]. In a healthy individual, the generation and excretion of uric acid are balanced, maintaining stable blood uric acid levels. However, increased factors contributing to uric acid production or impaired renal excretion can result in elevated blood uric acid levels [2]. In clinical practice, hyperuricemia is defined as a serum uric acid level of ≥ 7 mg/dL in males and ≥ 6 mg/dL in female [3]. The rise in living standards in recent years has led to an increasing occurrence of hyperuricemia, making it the second most prevalent metabolic disorder after diabetes and posing a significant threat to human health [4]. Statistical data reveals more than 12% increase in the prevalence of hyperuricemia in the United States between 1988 and 2016 [5]. Hyperuricemia is associated with the development of gout, urinary tract stones, and kidney damage [6]. Furthermore, emerging evidence suggests a close link between hyperuricemia and cardiovascular disease, diabetes, obesity, and other conditions [7, 8].

Oxidative stress is a pathological condition characterized by a disruption in the equilibrium between pro-oxidant and antioxidant factors, with a predominance of pro-oxidants. This disruption can result in cellular and tissue damage through oxidative mechanisms, leading to inflammation, cellular injury, and various diseases [9]. Besides dietary factors, the body’s oxidative stress status is also influenced by various lifestyle elements such as obesity, alcohol consumption, smoking, and physical activity [10–12]. .

The Oxidative Balance Score (OBS) is a refined indicator for evaluating an individual’s state of oxidative balance primarily constituted by dietary and lifestyle elements [13]. Elevated OBS typically suggests a predominance of antioxidants, surpassing pro-oxidants [13]. Numerous epidemiological investigations have demonstrated an inverse correlation between OBS and different inflammation-related diseases, including cardiovascular diseases, chronic kidney disease, as well as type 2 diabetes [14, 15]. However, current studies do not provide sufficient evidence to confirm the relationship between OBS and hyperuricemia. Our study aimed to perform a cross-sectional analysis utilizing data from the National Health and Nutrition Examination Survey (NHANES) between 2007 and 2018 to investigate the association between OBS, integrated with lifestyle and dietary components, and hyperuricemia.

## Materials and methodology

### Study design and population

**Fig. 1:**
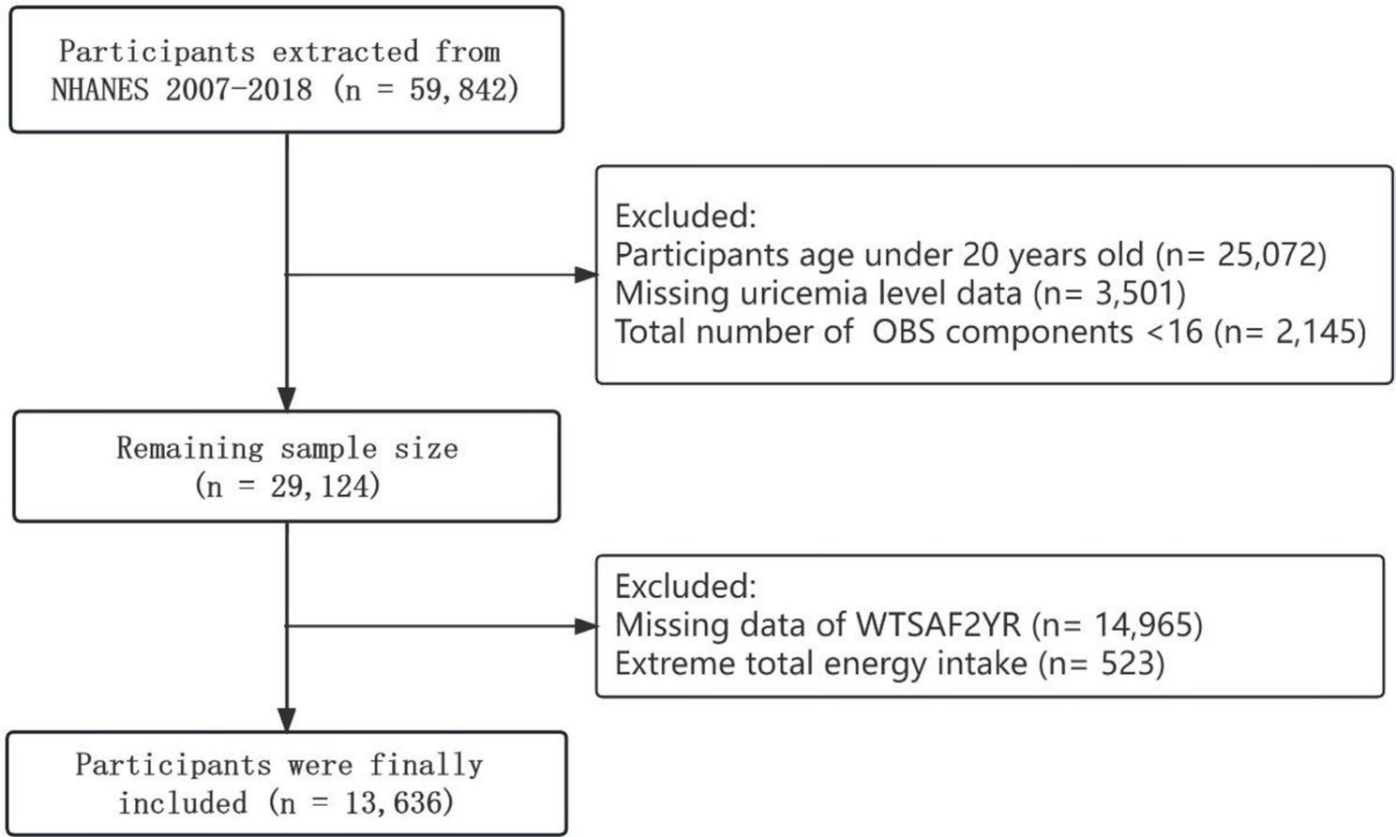
Flowchart showing the selection of the studied population

This study utilized data from 2007 to 2018 obtained from the NHANES, which conducted a national cross-sectional survey and employed a multistage stratified probability sample. These specific cycles were chosen due to the consistent measurement of the variables required, particularly the physical activity questionnaire (PAQ). Among the initially extracted 59,842 participants, we made exclusions for the following reasons: (1) younger than 20 years old (*n* = 25,072), (2) missing SUA level data (*n* = 3,501), (3) a total count of less than 16 out of the 20 components of the OBS (*n* = 2,145), (4) loss of WTSAF2YR data (*n* = 14,965), and (5) further exclusion of 1,714 participants with extreme energy intake (total energy intakes below 800 or above 4,200 kcal/day for males and below 500 or above 3,500 kcal/day for females) [16]. Ultimately, the study encompassed 13,636 participants (Fig. 1).

### Serum uric acid level and hyperuricemia

We defined the outcomes as serum uric acid (SUA) level and the prevalence of hyperuricemia to investigate their association with the oxidative balance score. Serum samples were collected from the participants and stored at -30℃ before being transmitted to the CDC/NCEH/DLS for examination. The SUA level was measured as part of the routine serum biochemistry profile using the Beckman Coulter UniCel® DxC800.

### Oxidative balance score

The Oxidative Balance Score (OBS) was determined by integrating 16 dietary factors and 4 lifestyle components, comprising 5 pro-oxidants and 15 antioxidants [17]. Dietary OBS, including dietary fiber, total fat, total folate, vitamins (B6, B12, C and E), niacin, carotene, riboflavin, calcium, iron, selenium, copper, magnesium, and zinc, which were collected from dietary recalls. The lifestyle OBS consists of four components: smoking, alcohol consumption, physical activity, and body mass index (BMI). Serum cotinine levels were used to quantify the smoking factor, which reflects both direct smoking and exposure to second-hand smoking. Alcohol consumption and BMI were categorized into three groups based on different gender. Total physical activity was quantified as the metabolic equivalent of task (MET) [18], calculated based on the accumulated time of transportation and moderate and vigorous activities per week. Except for alcohol consumption and BMI, the other OBS components were categorized by gender and divided into tertiles. Scores were assigned to antioxidants in tertiles 1 to 3 as 0, 1, and 2, respectively, while scores were assigned to pro-oxidants in the opposite order (Table S1). A higher OBS score indicates a more substantial antioxidant effect. This study included participants with a total count of at least 16 out of the 20 OBS components. In cases where OBS components were missing, the corresponding component was assigned a score of 0, regardless of its antioxidant or pro-oxidant nature.

### Covariates

Prior research and clinical considerations informed the selection of covariates. Demographic characteristics, such as age, race, gender, educational level, and poverty income ratio (PIR), were obtained through standardized household interviews. Age was divided into four categories, each representing a twenty-year range. Race was categorized as Hispanic, non-Hispanic white, non-Hispanic black, and other races. Educational level was classified as below, equal to, and above high school. Poverty was classified into three groups based on PIR (< 1.3, [1.3, 3.5), and ≥ 3.5). We also included commonly observed outcomes of metabolic disorders, namely cardiovascular disease (CVD), diabetes, chronic kidney disease (CKD), hypertension, as well as hyperlipidemia. Participants with CKD were defined by a questionnaire: “Ever been told by a doctor or other health professional that you had weak or failing kidneys?” or diagnosed by estimated Glomerular Filtration Rate (eGFR) < 60 mL/min·1.73 m^2^ and albumin-to-creatinine ratio (ACR) ≥ 30 mg/g [16]. The definition of the other comorbidities was collected in Table S2. Daily intake of energy, caffeine, protein, and sugar were also recruited in our study. Values of dietary intake in this study were summarized for each variable. In cases of missing data in the second recall, the average dietary intake from both recalls or sole data from the first 24-hour interview was utilized.

### Statistical analysis

According to the NHANES recommended sample weight on Fasting Subsample 2 Year MEC Weight (WTSAF2YR) records, sample weights of individuals were determined by WTSAF2YR/6. Nonnormally distributed continuous variables were described using median and interquartile range (IQR) to analyze baseline characteristics. Meanwhile, categorical variables were reported as sample counts and weighted percentages. To examine variations in variable characteristics among OBS groups (quartiles), we employed the Wilcoxon rank-sum test for continuous variables and the Rao-Scott chi-squared test for weighted percentages of categorical variables, comprehensively describing the entire population. To compare OBS with different outcomes (hyperuricemia and SUA level), weighted logistic and linear regression were employed. Model 1 represented the crude model, while Model 2 included adjustments for grouped age, gender, race, PIR, and dietary intake of energy, caffeine, protein, and sugar. Model 3 further adjusted for CKD, diabetes, CVDs, hypertension, and hyperlipidemia upon the adjustments in model 2. All regressions incorporated survey weights, and non-normally distributed continuous covariates were transformed using weighted quartiles. We further categorized OBS into dietary and lifestyle subtypes to explore their correlation with outcomes. Additionally, interaction analyses were conducted to assess potential interactions between each subgroup and OBS. Furthermore, a weighted restricted cubic spline (RCS) curve was performed to investigate the potentially nonlinear association between exposure and outcome. Sensitivity assessments were performed by iteratively removing each OBS component from model 3. Statistical analyses were two-sided, and *p* < 0.05 was considered statistically significant. All analyses were performed using R version 4.2.3, employing various packages such as *gtsummary, survey, rms, ggplot2*, and *forestplot*.

## Results

### Baseline characteristics

Our study included 6,516 males and 7,120 females. Table 1 presents the baseline characteristics of the participants, stratified by quartiles of total OBS. Individuals in the higher OBS quartile were characterized by older age and a higher proportion of non-Hispanic white ethnicity than those in the lower OBS quartile. In the higher quartile, we also observed an increase in the consumption of energy, caffeine, protein, and sugar, along with higher levels of education and wealth among individuals. However, the differences in gender and hypertension were not statistically significant. Moreover, as the total OBS increased, there was a gradual decline in the levels of SUA and the prevalence of hyperuricemia, along with co-existing diseases such as CKD, diabetes, hyperlipidemia, and CVDs. Table S3 presents additional baseline characteristics categorized according to the absence or presence of hyperuricemia. Individuals with hyperuricemia tended to be older and had lower OBS scores, including total OBS, life OBS, and dietary OBS. The hyperuricemia group also exhibited a more significant burden of comorbidities. The gender, educational level, and PIR showed no significant difference between the two groups.

**Table 1:** Baseline characteristics of participants according to the oxidative balance score’s quartile

Characteristic	Overall^1^, *N* = 206,933,8241	Q1^1^ [0,14), *N* = 206,933,823, *n* = 3986 (26%)	Q2^1^ [14,20), *N* = 206,933,823, *n* = 3549 (25%)	Q3^1^ [20,26), *N* = 206,933,823, *n* = 3214 (24%)	Q4^1^ ≥ 26, *N* = 206,933,823, *n* = 2887 (25%)	*p*-value^2^
**SUA level (mg/dL)**	5.40 (4.50, 6.30)	5.60 (4.60, 6.50)	5.50 (4.50, 6.50)	5.40 (4.50, 6.34)	5.10 (4.30, 6.10)	**< 0.001**
**Age**	47.00 (33.00, 60.00)	45.00 (31.00, 58.00)	46.00 (31.00, 60.00)	47.00 (33.00, 60.00)	52.00 (37.00, 63.00)	**< 0.001**
**Energy(kcal)**	1,971.00 (1,522.50, 2,507.50)	1,576.50 (1,223.00, 1,976.94)	1,962.00 (1,557.72, 2,403.00)	2,145.72 (1,699.50, 2,706.61)	2,278.00 (1,793.51, 2,866.55)	**< 0.001**
**Caffeine(mg)**	121.50 (39.50, 235.50)	103.00 (34.50, 216.00)	122.50 (42.00, 239.00)	129.50 (43.00, 232.50)	138.00 (43.00, 247.50)	**< 0.001**
**Protein(g)**	76.51 (57.84, 98.67)	56.96 (44.70, 71.86)	75.17 (59.25, 92.85)	85.41 (66.89, 107.97)	92.66 (73.17, 118.15)	**< 0.001**
**Sugar(g)**	96.09 (63.60, 137.27)	78.81 (50.16, 117.43)	90.73 (61.54, 132.51)	101.42 (68.98, 141.83)	113.47 (79.64, 150.03)	**< 0.001**
**Gender**						0.11
Female	7,120.00 (52.46%)	2,066.00 (52.70%)	1,792.00 (50.32%)	1,718.00 (52.67%)	1,544.00 (54.16%)	
Male	6,516.00 (47.54%)	1,920.00 (47.30%)	1,757.00 (49.68%)	1,496.00 (47.33%)	1,343.00 (45.84%)	
**Race**						**< 0.001**
Hispanic	3,588.00 (14.27%)	1,052.00 (15.25%)	981.00 (15.57%)	924.00 (15.71%)	631.00 (10.52%)	
Non-Hispanic white	5,809.00 (67.26%)	1,494.00 (61.00%)	1,403.00 (63.92%)	1,360.00 (67.88%)	1,552.00 (76.48%)	
Non-Hispanic black	2,681.00 (10.84%)	1,118.00 (17.59%)	720.00 (11.53%)	521.00 (8.74%)	322.00 (5.27%)	
other races	1,558.00 (7.63%)	322.00 (6.16%)	445.00 (8.98%)	409.00 (7.66%)	382.00 (7.74%)	
**Education**						**< 0.001**
< High school	3,315.00 (15.70%)	1,311.00 (23.34%)	905.00 (16.64%)	685.00 (13.96%)	414.00 (8.58%)	
High school	3,063.00 (22.73%)	1,060.00 (29.37%)	839.00 (24.79%)	645.00 (19.27%)	519.00 (17.21%)	
> High school	7,246.00 (61.55%)	1,609.00 (47.23%)	1,803.00 (58.54%)	1,881.00 (66.75%)	1,953.00 (74.20%)	
missing	12.00 (0.03%)	6.00 (0.06%)	2.00 (0.03%)	3.00 (0.02%)	1.00 (0.01%)	
**Poverty Ratio**						**< 0.001**
<1.3	3,963.00 (20.22%)	1,520.00 (30.00%)	1,066.00 (21.36%)	823.00 (17.04%)	554.00 (12.14%)	
≥ 1.3,<3.5	4,742.00 (34.09%)	1,405.00 (36.52%)	1,279.00 (36.62%)	1,107.00 (33.93%)	951.00 (29.20%)	
≥ 3.5	3,711.00 (39.02%)	690.00 (26.68%)	873.00 (35.05%)	988.00 (41.98%)	1,160.00 (52.81%)	
missing	1,220.00 (6.67%)	371.00 (6.79%)	331.00 (6.98%)	296.00 (7.05%)	222.00 (5.86%)	
**CKD**						< 0.001
No	12,947.00 (96.37%)	3,712.00 (95.00%)	3,377.00 (96.28%)	3,072.00 (96.67%)	2,786.00 (97.58%)	
Yes	689.00 (3.63%)	274.00 (5.00%)	172.00 (3.72%)	142.00 (3.33%)	101.00 (2.42%)	
**Diabetes**						< 0.001
No	10,982.00 (85.94%)	3,084.00 (83.31%)	2,834.00 (85.71%)	2,638.00 (86.56%)	2,426.00 (88.29%)	
Yes	2,654.00 (14.06%)	902.00 (16.69%)	715.00 (14.29%)	576.00 (13.44%)	461.00 (11.71%)	
**Hypertension**						0.053
No	11,277.00 (86.60%)	3,190.00 (85.25%)	2,932.00 (86.15%)	2,722.00 (87.73%)	2,433.00 (87.33%)	
Yes	2,359.00 (13.40%)	796.00 (14.75%)	617.00 (13.85%)	492.00 (12.27%)	454.00 (12.67%)	
**Hyperlipidemia**						< 0.001
No	5,022.00 (37.66%)	1,333.00 (32.48%)	1,288.00 (38.10%)	1,206.00 (37.57%)	1,195.00 (42.63%)	
Yes	8,613.00 (62.34%)	2,653.00 (67.52%)	2,260.00 (61.89%)	2,008.00 (62.43%)	1,692.00 (57.37%)	
missing	1.00 (0.00%)	0.00 (0.00%)	1.00 (0.00%)	0.00 (0.00%)	0.00 (0.00%)	
**CVD**						0.029
No	12,086.00 (90.86%)	3,412.00 (89.24%)	3,158.00 (90.17%)	2,907.00 (92.29%)	2,609.00 (91.82%)	
Yes	1,549.00 (9.14%)	573.00 (10.76%)	391.00 (9.83%)	307.00 (7.71%)	278.00 (8.18%)	
missing	1.00 (0.00%)	1.00 (0.00%)	0.00 (0.00%)	0.00 (0.00%)	0.00 (0.00%)	
**Hyperuricemia**						< 0.001
No	10,577.00 (78.31%)	2,913.00 (74.30%)	2,741.00 (77.10%)	2,551.00 (78.18%)	2,372.00 (83.75%)	
Yes	3,059.00 (21.69%)	1,073.00 (25.70%)	808.00 (22.90%)	663.00 (21.82%)	515.00 (16.25%)	

SUA, serum uric acid; CKD, chronic kidney disease; CVD, cardiovascular disease; Q, quartile

Q1: 0 ≤ Total OBS<14; Q2: 14 ≤ Total OBS<20; Q3: 20 ≤ Total OBS<26; Q4 Total OBS ≥ 26.

1 Median (IQR); n (unweighted) (%); N (weighted) (%)

2 Wilcoxon rank-sum test for complex survey samples; chi-squared test with Rao & Scott’s second-order correction

### Correlation between OBS and uricemia

Unilinear and unilogistic regression models were performed to investigate the association between OBS and uricemia (Table S4). All three definitions of OBS presented a negative correlation with abnormal uricemia, defined by elevated serum uric acid (SUA) levels and a higher prevalence of hyperuricemia. Dietary intake, including energy, protein, and sugar, negatively correlated with these outcomes, while caffeine intake exhibited the opposite trend. Factors such as advanced age, higher socioeconomic status, and comorbidities were correlated with elevated levels of SUA and an increased incidence of hyperuricemia. Furthermore, multivariate linear models and logistic regression were applied, as presented in Table 2. Education was excluded as a covariate in the adjusted regression models due to its lack of significance in both unilinear and unilogistic regressions. In the fully adjusted Model 3, a higher continuous total OBS showed a significant inverse correlation with the incidence of hyperuricemia (odds ratio [OR]: 0.97, 95% confidence interval [95% CI]: 0.96 to 0.98). When compared to the reference category of the first OBS quartile, the OR (95% CI) for the third OBS quartile was 0.80 (0.67 to 0.97), and for the fourth OBS quartile, it was 0.55 (0.44 to 0.70). The observed trend was highly significant (*p* < 0.001), indicating a 45% decrease in the probability of hyperuricemia for each increase from the first to the fourth OBS quartile. Furthermore, the linear regression analysis indicated a negative correlation between total OBS and SUA levels. Even after adjusting for all covariates, a significant association between higher continuous total OBS and lower levels of SUA persisted (β: -0.02, 95% CI: -0.03 to -0.02). Similarly, when considering total OBS quartiles, the fourth quartile exhibited a β coefficient of -0.4 (95% CI: -0.50 to -0.29) in comparison to the reference category of the first quartile.

**Table 2:** The relationship between OBS and uricemia

	Model1			Model2			Model3		
Characteristic	OR^1^	95% CI^1^	*P*-value	OR^1^	95% CI^1^	*P*-value	OR^1^	95% CI^1^	*P*-value
Total OBS continuous	0.97	(0.96, 0.98)	< 0.001	0.96	(0.95, 0.97)	< 0.001	0.97	(0.96, 0.98)	< 0.001
Total OBS quantile									
Q1	ref.	ref.		ref.	ref.		ref.	ref.	
Q2	0.86	(0.75, 0.98)	0.03	0.82	(0.70, 0.96)	0.012	0.86	(0.74, 1.01)	0.069
Q3	0.81	(0.69, 0.94)	0.006	0.75	(0.63, 0.90)	0.003	0.8	(0.67, 0.97)	0.023
Q4	0.56	(0.46, 0.68)	< 0.001	0.49	(0.39, 0.62)	< 0.001	0.55	(0.44, 0.70)	< 0.001
p for trend	0.67	(0.59, 0.76)	< 0.001	0.61	(0.52, 0.71)	< 0.001	0.66	(0.56, 0.78)	< 0.001

OR, odds ratio; CI, confidence intervals; OBS, oxidative balance score

Q1: 0 ≤ Total OBS<14; Q2: 14 ≤ Total OBS<20; Q3: 20 ≤ Total OBS<26; Q4: Total OBS ≥ 26.

The model 1 was the crude model

The model 2 was adjusted by age, gender, race, poverty-to-income ratio, energy intake, caffeine intake, protein intake and sugar intake

The model 3 was adjusted by age, gender, race/ethnicity, poverty-to-income ratio, CKD, diabetes, hypertension, hyperlipidemia, CVD, energy intake, caffeine intake, protein intake and sugar intake

### Relationship between dietary/ lifestyle OBS and uricemia

We further explored the association between OBS and uricemia by categorizing OBS into dietary and lifestyle OBS, as presented in Table 3. Both dietary and lifestyle OBS subtypes exhibited statistically significant associations with uricemia. Higher levels of continuous dietary and lifestyle OBS consistently showed correlations with decreased SUA levels and a reduced incidence of hyperuricemia. In the fully adjusted Model 3, the fourth quartile of dietary OBS significantly impacted hyperuricemia (OR: 0.68, 95% CI: 0.53 to 0.87) and SUA levels (β: -0.27, 95% CI: -0.38 to -0.16) compared to the lowest quartile. Similarly, lifestyle OBS also demonstrated a persistent effect, with the fourth quartile indicating a negative correlation with hyperuricemia (OR: 0.39, 95% CI: 0.33 to 0.48) and SUA levels (β: -0.54, 95% CI: -0.62 to -0.47).

**Table 3:** The relationship between dietary/lifestyle OBS and uricemia

	Model1			Model2			Model3		
Characteristic	OR^1^	95% CI^1^	*P*-value	OR^1^	95% CI^1^	*P*-value	OR^1^	95% CI^1^	*P*-value
Dietary OBS	0.98	0.97, 0.99	< 0.001	0.98	0.96, 0.99	< 0.001	0.98	0.97, 0.99	0.001
**Dietary OBS quantile**									
Q1	ref.	ref.		ref.	ref.		ref.	ref.	
Q2	0.91	0.79, 1.05	0.2	0.89	0.76, 1.04	0.14	0.92	0.78, 1.08	0.3
Q3	0.89	0.77, 1.03	0.12	0.86	0.73, 1.02	0.087	0.9	0.75, 1.07	0.2
Q4	0.66	0.55, 0.81	< 0.001	0.61	0.48, 0.78	< 0.001	0.68	0.53, 0.87	0.003
p for trend	0.76	0.67, 0.86	< 0.001	0.72	0.61, 0.84	< 0.001	0.77	0.65, 0.91	0.002
Life OBS	0.75	0.72, 0.78	< 0.001	0.75	0.71, 0.78	< 0.001	0.77	0.74, 0.81	< 0.001
**Life OBS quantile**									
Q1	ref.	ref.		ref.	ref.		ref.	ref.	
Q2	0.66	0.57, 0.76	< 0.001	0.64	0.55, 0.75	< 0.001	0.67	0.58, 0.79	< 0.001
Q3	0.43	0.37, 0.51	< 0.001	0.43	0.36, 0.50	< 0.001	0.47	0.39, 0.55	< 0.001
Q4	0.24	0.18, 0.31	< 0.001	0.23	0.18, 0.31	< 0.001	0.28	0.22, 0.37	< 0.001
p for trend	0.35	0.29, 0.41	< 0.001	0.34	0.28, 0.42	< 0.001	0.39	0.33, 0.48	< 0.001

OR, odds ratio; CI, confidence intervals; OBS, oxidative balance score

Dietary OBS: Q1: 0 ≤ Dietary OBS<10; Q2: 10 ≤ Dietary OBS<16; Q3: 16 ≤ Dietary OBS<22; Q4: Dietary OBS ≥ 22.

Life OBS: Q1: 0 ≤ Life OBS<3; Q2: 3 ≤ Life OBS<4; Q3: 4 ≤ Life OBS<6; Q4: Life OBS ≥ 6.

The model 1 was the crude model

The model 2 was adjusted by age, gender, race, poverty-to-income ratio, energy intake, caffeine intake, protein intake and sugar intake

The model 3 was adjusted by age, gender, race/ethnicity, poverty-to-income ratio, CKD, diabetes, hypertension, hyperlipidemia, CVD, energy intake, caffeine intake, protein intake and sugar intake

### Subgroup analysis

**Fig. 2:**
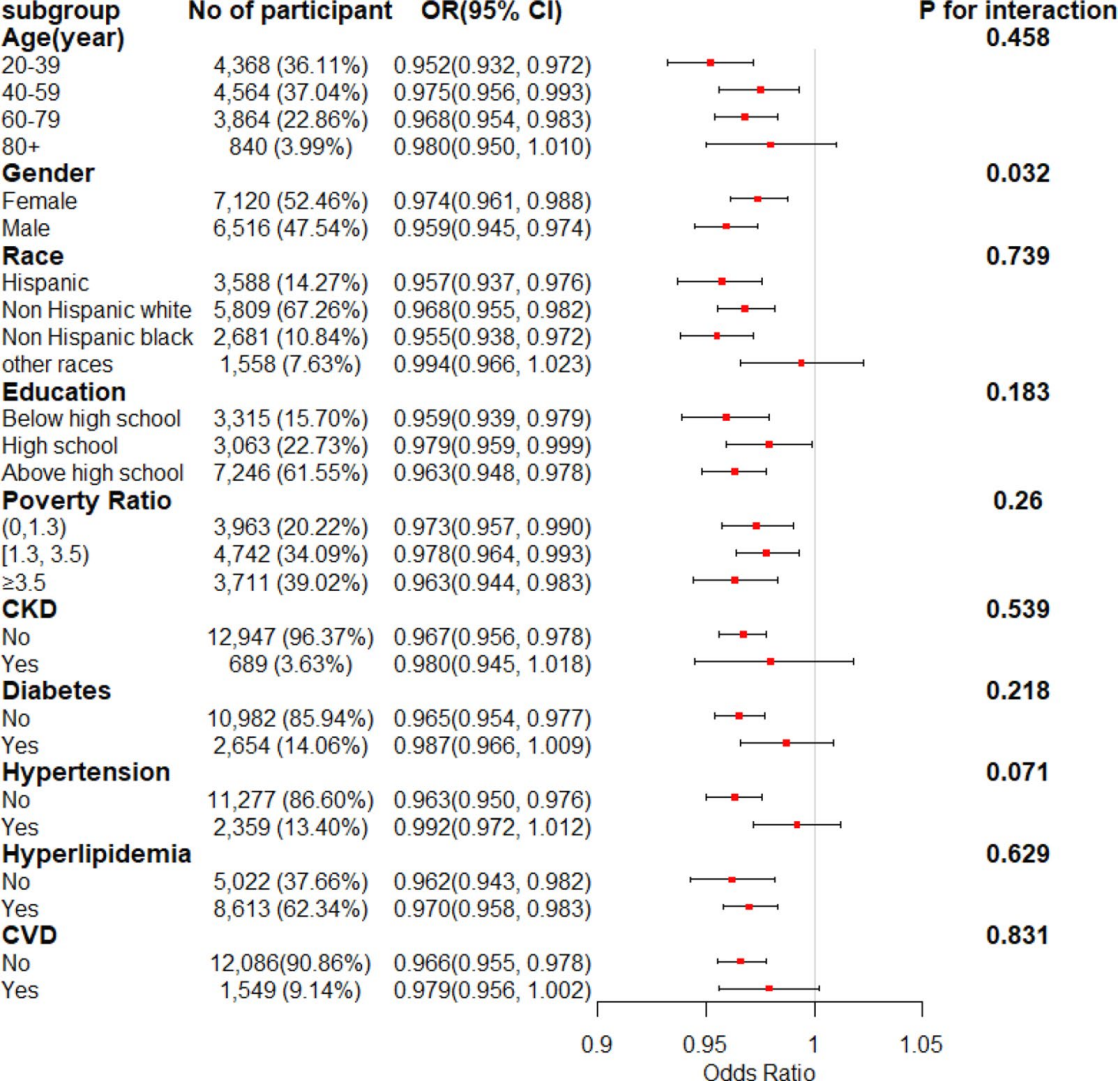
The relationship between Total OBS and hyperuricemia in various subgroups

Fig.2 illustrates the association between total OBS and hyperuricemia, analyzed through fully adjusted multivariate logistic regression in various subgroups stratified by age, race, gender, education levels, PIR, and comorbidities. A significant inverse association between total OBS and hyperuricemia incidence was observed in most subgroups. Nevertheless, no substantial association was detected between total OBS and hyperuricemia among individuals aged 80 years and older, those belonging to other racial backgrounds, or those with comorbidities such as CKD, cardiovascular diseases (CVDs), diabetes, and hypertension. The interaction analysis revealed a statistically significant difference in the gender subgroup (P for interaction = 0.032), whereas no other subgroups exhibited statistical significance

### Restricted cubic splines

**Fig. 3:**
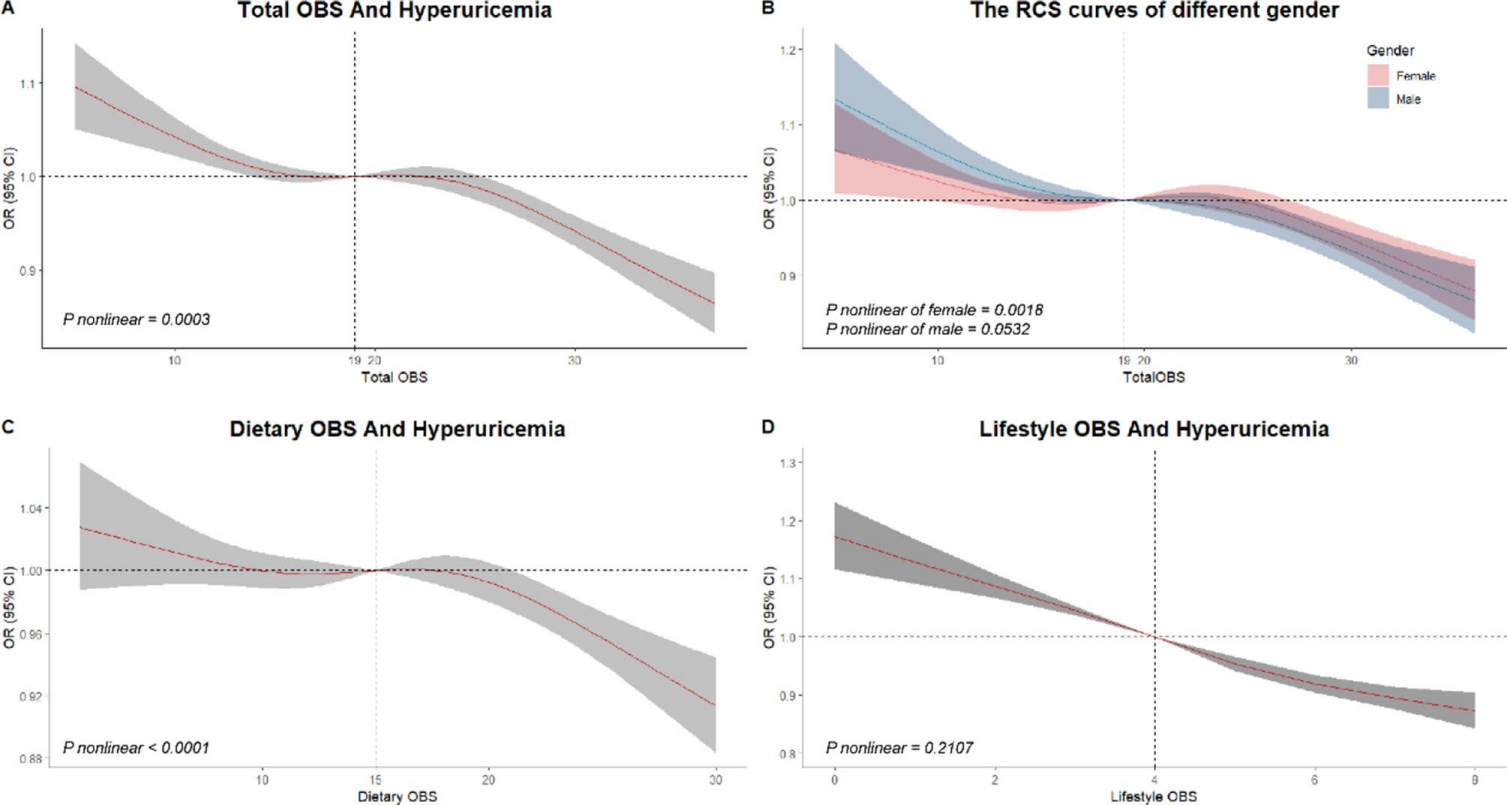
RCS curves of the relationships between three definitions of OBS and hyperuricemia

Restricted Cubic Splines (RCS) curves were employed to investigate the relationships between three definitions of OBS and hyperuricemia, adjusting for all relevant covariates. A statistically significant nonlinear relationship was found between total OBS and hyperuricemia (P nonlinear = 0.0003, Fig. 3A). Consistently, the odds ratio for hyperuricemia decreased with increasing total OBS, and this trend was constant in females (p for nonlinear < 0.0001, Fig. 3B). The overall trend of the curve showed a decline with a relatively plateau section around 19, both in female subgroups and the general population. In contrast, no statistically significant nonlinear correlation was found between total OBS and hyperuricemia in male subgroups. Furthermore, dietary OBS exhibited a significant inverse and nonlinear relationship with the incidence of hyperuricemia (P nonlinear < 0.0001, Fig. 3C). In contrast, no such relationship was observed with lifestyle OBS (P nonlinear = 0.2107, Fig. 3D). Although the results of the restricted cubic splines analysis showed slight variations, the overall patterns between the exposures and outcome remained stable across the graphs.

### Additional analysis

**Fig. 4:**
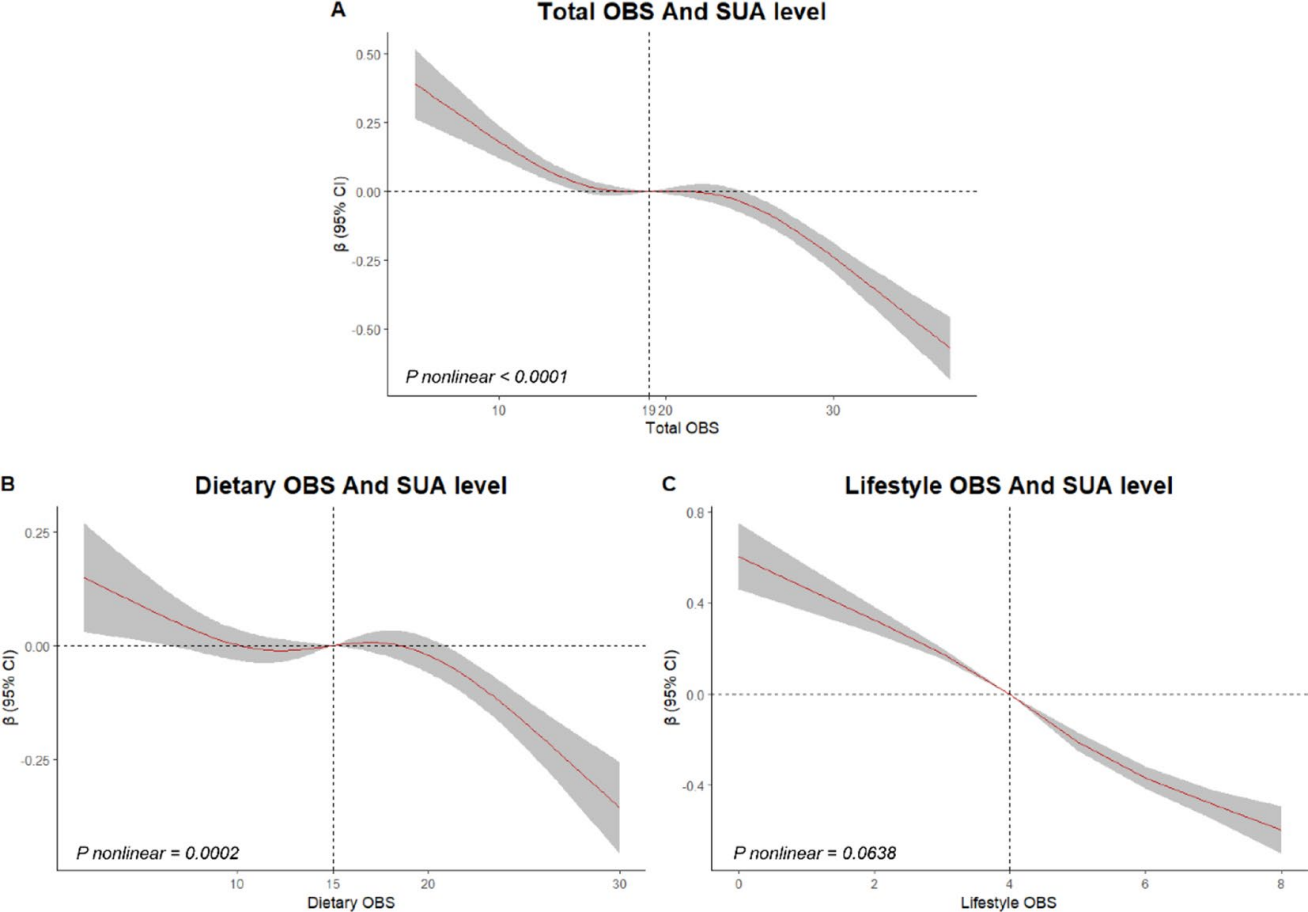
RCS curves of the relationships between three distinct OBS types and SUA levels

The present study further investigates the association between three distinct types of OBS and SUA levels using RCS curves. Consistent with our previous findings, the results depicted in Fig. 4 illustrate a significant negative nonlinear correlation between SUA levels and both total OBS (P nonlinear < 0.0001, Fig. 4A) and dietary OBS (P nonlinear = 0.0002, Fig. 4B). Conversely, no statistically significant nonlinear correlation was presented between lifestyle OBS and SUA levels (P nonlinear = 0.0638, Fig. 4C). These findings align with our former analysis, which focused on hyperuricemia as the primary outcome of interest. Additionally, a sensitivity analysis involving the iterative removal of individual OBS components revealed no significant impact on the association with SUA level and hyperuricemia outcomes, as detailed in Table S5.

## Discussion

This study observed that higher total OBS was correlated with a decreased level of SUA and decreased incidence of hyperuricemia, with a significant trend across OBS quartiles. The negative correlation persisted even after adjusting for various covariates, indicating a 45% decrease in the probability of hyperuricemia from the first to the fourth OBS quartile. Further analysis into dietary OBS and lifestyle OBS revealed consistent associations, with higher quartiles demonstrating a significant impact on hyperuricemia and SUA levels. Subgroup analysis revealed a notable gender-based interaction, indicating a statistically significant difference in the association between total OBS and hyperuricemia across different gender groups. Moreover, Restricted Cubic Splines (RCS) analysis highlighted a significantly negative nonlinear association between total OBS and hyperuricemia, particularly in female subgroups. The overall trend declined with a relatively plateau section around a total OBS of 19. Interestingly, lifestyle OBS did not exhibit a significant nonlinear relationship with hyperuricemia in the RCS analysis. Finally, sensitivity analysis confirmed the robust correlation between OBS and uricemia.

In recent years, significant advancements have been made in comprehending the intricate association between uric acid and oxidative stress and their underlying molecular mechanisms. A study conducted on chicken embryonic cardiomyocytes revealed that physiological levels of uric acid can mitigate oxidative stress, enhance superoxide dismutase activity, and inhibit reactive oxygen species (ROS) formation [19]. SUA levels exhibit dual characteristics, acting as a pro-oxidant and an antioxidant. Extracellular uric acid primarily functions as an antioxidant, acting as a scavenger, and has demonstrated certain advantages in neurological disorders [20]. Conversely, intracellular uric acid predominantly assumes a pro-oxidative role by activating mechanisms such as NADPH oxidase [21]. It can diminish nitric oxide levels in endothelial cells, trigger peroxynitrite-mediated lipid oxidation, and activate pro-inflammatory biomarkers [20, 22]. Furthermore, research suggests hyperuricemia-induced oxidative stress may contribute to endothelial cell aging and apoptosis [23, 24]. Hence, a comprehensive assessment of the interplay between oxidative stress and hyperuricemia holds particular significance.

Diet plays a significant role in hyperuricemia, as increased purine intake can lead to elevated uric acid production [25]. Dietary modifications are often recommended as an adjunct to gout treatment to reduce uric acid levels [26]. Consequently, there is considerable interest in exploring the potential impact of diet on managing hyperuricemia, with a substantial body of literature investigating the relationship between diet and hyperuricemia. Plant-based diets, Mediterranean dietary score, and Dietary Approaches to Stop Hypertension (DASH) diet have been examined concerning hyperuricemia [27, 28]. Research has demonstrated that dietary antioxidants, such as dietary fiber, zinc, magnesium, and vitamin D, can inhibit the development of hyperuricemia [29–32]. Lifestyle factors have also been investigated, with studies confirming a positive correlation between higher BMI and hyperuricemia [33]. Adequate exercise has been found to reduce the occurrence of hyperuricemia to some extent [34]. We further categorized the total OBS into lifestyle and dietary OBS and analyzed their associations with hyperuricemia. Our findings revealed that higher dietary and lifestyle OBS were linked to lower SUA levels and a reduced prevalence of hyperuricemia. In Model 3, after fully adjusting for confounding variables, the fourth quartile of dietary OBS significantly impacted hyperuricemia and SUA levels compared to the lowest quartile. Interestingly, lifestyle OBS had a more significant impact on hyperuricemia per unit than dietary OBS.

In our subgroup analysis, we observed higher SUA levels in both non-Hispanic white and black populations, with men exhibiting a higher incidence of hyperuricemia compared to women. These findings suggest that variations in genetic susceptibility, dietary habits, or other lifestyle factors may contribute to the observed variability in the relationship between hyperuricemia and OBS across different subgroups. According to previous research from NHANES, the incidence of hyperuricemia in men and women was 20.2% and 20.0% in 2015, respectively [5]. The incidence rate of hyperuricemia remained relatively stable from 2007 to 2016. In Ireland, from 2006 to 2014, the incidence of hyperuricemia increased from 19.7 to 25.0% in males and from 20.5 to 24.1% in females [35]. Furthermore, most epidemiological studies indicate a higher incidence of hyperuricemia in wealthier countries [36, 37]. Our study also revealed that individuals with hyperuricemia had higher levels of wealth, which may be attributed to increased income, higher energy intake, and a diet rich in purine-containing foods. Regarding age subgroups, individuals over 60 exhibited higher SUA levels in our study, possibly due to reduced physical activity and slower metabolism, leading to increased uric acid levels.

Furthermore, higher SUA levels and elevated incidence of hyperuricemia were observed in various subgroups of chronic diseases, including diabetes, hypertension, cardiovascular disease (CVD), chronic kidney disease (CKD), and hyperlipidemia populations. However, no significant correlation was found between OBS and hyperuricemia across these subgroups with chronic diseases except for hyperlipidemia. The results can be attributed to the intricate interactions among these diseases, oxidative stress, and hyperuricemia. Metabolic disorders, including hyperuricemia, hypertension, hyperlipidemia, and diabetes, among others, are closely linked to insulin resistance [38]. Previous studies have confirmed that oxidative stress and inflammation may serve as the pathological and physiological onset of insulin resistance. Hyperuricemia elevates the phosphorylation of insulin receptor substrate 1 (IRS1) and inhibits phosphorylation of Akt, while elevated levels of reactive oxygen species (ROS) can induce insulin resistance [39]. Moreover, studies have confirmed a close relationship between hyperuricemia and hypertension, although the exact mechanism is still not fully understood. Hyperinsulinemia caused by insulin resistance enhances sodium reabsorption by the kidneys, which may contribute to hypertension [40]. The potential mechanisms of uric acid-induced coronary heart disease include endothelial damage in large and micro-vessels, as well as the involvement of hyperuricemia in the production of various inflammatory mediators [41]. However, more detailed mechanisms require further in-depth research. Overall, the complex interplay between oxidative stress, inflammation, insulin resistance, and hyperuricemia highlight the need for further investigation into the underlying mechanisms and their implications in various chronic diseases.

### Strengths and limitations

Our study possesses several strengths. To begin with, we utilized a large-scale national representative sample, enhancing the generalizability of our findings. Additionally, we adopted a comprehensive approach by considering both dietary and lifestyle factors, providing a thorough evaluation of pro-oxidative and antioxidant exposure through the OBS. Yet, there are still several limitations. Firstly, the cross-sectional survey restricts our ability to conduct causal relationships between OBS, hyperuricemia, and related chronic diseases. Longitudinal studies would be valuable in further investigating these associations. Another limitation is the absence of specific data on oxidative and inflammatory markers, such as malondialdehyde (MDA), nitric oxide (NO), and other related inflammatory factors in the NHANES database. Including these biomarkers could offer a more comprehensive understanding of the underlying mechanisms. Besides, our study relied on self-reported data and 24-hour dietary recall, introducing the potential for recall bias. Additionally, our findings may not apply directly to all individuals with hyperuricemia, as the study population represented the general population. Future research should consider incorporating more objective measures of oxidative stress and inflammation biomarkers to address these limitations to obtain accurate and precise results. As well, longitudinal studies would help support our results and illuminate the potential mechanisms behind the observed relationships. While this study provides valuable insights into the relationship between OBS and hyperuricemia, further research is warranted to overcome the limitations and establish a more robust understanding of these associations.

## Conclusions

Our study highlighted the significant negative association between OBS and the prevalence of hyperuricemia, as well as SUA levels. This robust negative correlation persisted across various demographic subgroups, emphasizing the potential universal relationship between OBS and hyperuricemia. Notably, both dietary and lifestyle OBS demonstrated consistent and independent associations with reduced SUA levels and a lower prevalence of hyperuricemia. Nonlinear analysis further revealed a compelling negative trend between total OBS and hyperuricemia, particularly pronounced in female subgroups. Our study collectively emphasized the underlying negative relationship between OBS and hyperuricemia, providing valuable insights for future interventions and dietary strategies. However, further longitudinal studies and comprehensive biomarker assessments are necessary to elucidate the causal mechanisms and validate the long-term impact of OBS on hyperuricemia.

### Electronic supplementary material

Below is the link to the electronic supplementary material.

Supplementary Material 1

## Data Availability

No datasets were generated or analysed during the current study.
