# Supplementary material for: Nonlinear relationship between oxidative balance score and hyperuricemia: analyses of NHANES 2007–2018
Source: Nutr J. 2024 May 4;23:48. doi: 10.1186/s12937-024-00953-1 (PMC11069158; doi:10.1186/s12937-024-00953-1)
Supplement: Supplementary file 1 — Supplementary Material 1 [file 12937_2024_953_MOESM1_ESM.docx]

Table S1 Ingredients that make up the oxidative balance score.

| OBS components | Property | Female | | | Male | | |
| --- | --- | --- | --- | --- | --- | --- | --- |
|  |  | 0 | 1 | 2 | 0 | 1 | 2 |
| **Dietary OBS** |  |  |  |  |  |  |  |
| Dietary fiber(g/d) | A | ＜11.45 | 11.45-17.40 | ≥17.40 | ＜13.50 | 13.50-20.80 | ≥20.80 |
| Carotene (RE/d) | A | ＜616.00 | 616.00-2184.00 | ≥2184.00 | ＜601.50 | 601.50-1890.00 | ≥1890.00 |
| Vitamin B6(mg/d) | A | ＜1.53 | 1.53-2.98 | ≥2.98 | ＜1.99 | 1.99-3.46 | ≥3.46 |
| Vitamin B12(mcg/d) | A | ＜3.45 | 3.45-8.71 | ≥8.71 | ＜4.46 | 4.45-9.69 | ≥9.69 |
| Riboflavin (mg/d) | A | ＜1.64 | 1.64-2.73 | ≥2.73 | ＜2.02 | 2.012-3.19 | ≥3.19 |
| Total Folate (mcg/d) | A | ＜259.50 | 259.50-389.50 | ≥389.50 | ＜330.50 | 330.50-495.00 | ≥495.00 |
| Niacin (mg/d) | A | ＜19.25 | 19.25-30.64 | ≥30.64 | ＜26.31 | 26.31-39.34 | ≥39.34 |
| Vitamin C (mg/d) | A | ＜52.40 | 52.40-132.05 | ≥132.05 | ＜49.45 | 49.45-132.50 | ≥132.50 |
| Vitamin E (ATE) (mg/d) | A | ＜5.29 | 5.29-8.29 | ≥8.29 | ＜6.37 | 6.37-9.92 | ≥9.92 |
| Calcium (mg/d) | A | ＜750.50 | 750.50-1206.00 | ≥1206.00 | ＜850.00 | 850.00-1308.00 | ≥1308.00 |
| Magnesium (mg/d) | A | ＜225.00 | 225.00-322.00 | ≥322.00 | ＜277.00 | 277.00-391.00 | ≥391.00 |
| Zinc (mg/d) | A | ＜8.43 | 8.43-14.5 | ≥14.5 | ＜11.28 | 11.28-17.76 | ≥17.76 |
| Copper (mg/d) | A | ＜0.95 | 0.95-1.50 | ≥1.50 | ＜1.12 | 1.1215-1.73 | ≥1.73 |
| Selenium (mcg/d) | A | ＜82.15 | 82.15-118.75 | ≥118.75 | ＜113.05 | 113.05-163.55 | ≥163.55 |
| Iron (mg/d) | P | ≥17.09 | 10.73-17.09 | ＜10.73 | ≥19.54 | 13.14-19.54 | ＜13.14 |
| Total fat (gm/d) | P | ≥77.54 | 54.15-77.54 | ＜54.15 | ≥104.58 | 72.01-104.58 | ＜72.01 |
|  |  |  |  |  |  |  |  |
| **Lifestyle OBS** |  |  |  |  |  |  |  |
| Physical activity  (MET-minutes/week) | A | ＜280.00 | 280.00-1920.00 | ≥1920.00 | ＜960.00 | 960.00-4320.00 | ≥4320.00 |
| cotinine(ng/mL) | P | ≥0.08 | 0.01-0.08 | ＜0.01 | ≥0.99 | 0.02-0.99 | ＜0.02 |
| Alcohol(g/d) | P | ≥15 | (0, 15) | non | ≥30 | (0, 30) | non |
| Body mass index(kg/m2) | P | ≥30 | [25, 30) | ＜25 | ≥30 | [25, 30) | ＜25 |

A stood for the antioxidant, P for the pro-oxidant, RE for the retinal equivalent, ATE for the alpha-tocopherol equivalent, and MET for the metabolic equivalent.

Table S2 The definition of the other comorbities.

Diabetes

1) Medical diagnosis of diabetes as recorded by the patient’s healthcare provider or current use of insulin or diabetic pills.

2) or glycohemoglobin A1c (HbA1c) level higher than 6.5%.

3) or fasting blood glucose level equal to or higher than 7.0 mmol/L.

4) or random blood glucose level equal to or higher than 11.1 mmol/L.

Hypertension

1) average systolic blood pressure ≥140 mmHg or average diastolic blood pressure ≥90 mmHg by the mean value of at least three times of measurement

2) or use of anti-hypertensive medications

3) or self-reported diagnosis of hypertension.

Hyperlipidemia

1) Triglyceride levels equal to or greater than 150 mg/dL

2) or hypercholesterolemia:

a. TC levels equal to or greater than 200 mg/dL,

b. or LDL-C levels equal to or greater than 130 mg/dL,

c. or HDL-C levels less than 40 mg/dL for males and less than 50 mg/dL for females.

Cardiovascular diseases (CVDs)

congestive heart failure, coronary heart disease, heart attack, angina, or stroke.

Table S3 Additional baseline characteristics based on the presence or absence of hyperuricemia.

| Characteristic | Overall,  N = 206,933,8241,  n = 13,636 | Non hyperuricemia,  N = 162,040,2701,  n = 10577 (78%)^1^ | hyperuricemia,  N = 44,893,5531,  n = 3059 (22%)^1^ | p-value^2^ |
| --- | --- | --- | --- | --- |
| Total OBS | 20.00 (14.00, 26.00) | 21.00 (15.00, 27.00) | 19.00 (13.00, 25.00) | <0.001 |
| Dietary OBS | 16.00 (10.00, 22.00) | 16.00 (10.00, 22.00) | 15.00 (9.00, 21.00) | <0.001 |
| Lifestyle OBS | 4.00 (3.00, 6.00) | 5.00 (4.00, 6.00) | 4.00 (3.00, 5.00) | <0.001 |
| Age | 47.00 (33.00, 60.00) | 46.00 (33.00, 59.00) | 53.00 (36.00, 65.00) | <0.001 |
| Energy(kcal) | 1,971.00 (1,522.50, 2,507.50) | 1,990.00 (1,547.50, 2,529.00) | 1,890.00 (1,458.00, 2,419.55) | <0.001 |
| Caffeine(mg) | 121.50 (39.50, 235.50) | 121.50 (37.00, 234.00) | 123.98 (48.00, 240.50) | 0.2 |
| Protein(g) | 76.51 (57.84, 98.67) | 76.96 (58.33, 99.43) | 74.40 (56.43, 95.76) | 0.011 |
| Sugar(g) | 96.09 (63.60, 137.27) | 97.79 (65.62, 138.77) | 87.60 (56.32, 131.53) | <0.001 |
| Gender |  |  |  | 0.3 |
| Female | 7,120.00 (52.46%) | 5,457.00 (52.12%) | 1,663.00 (53.67%) |  |
| Male | 6,516.00 (47.54%) | 5,120.00 (47.88%) | 1,396.00 (46.33%) |  |
| Race |  |  |  | <0.001 |
| Hispanic | 3,588.00 (14.27%) | 2,974.00 (15.28%) | 614.00 (10.61%) |  |
| Non-Hispanic white | 5,809.00 (67.26%) | 4,431.00 (66.65%) | 1,378.00 (69.46%) |  |
| Non-Hispanic black | 2,681.00 (10.84%) | 1,961.00 (10.42%) | 720.00 (12.34%) |  |
| other races | 1,558.00 (7.63%) | 1,211.00 (7.64%) | 347.00 (7.58%) |  |
| Education |  |  |  | 0.4 |
| ＜High school | 3,315.00 (15.70%) | 2,607.00 (15.88%) | 708.00 (15.04%) |  |
| High school | 3,063.00 (22.73%) | 2,335.00 (22.45%) | 728.00 (23.71%) |  |
| ＞High school | 7,246.00 (61.55%) | 5,627.00 (61.64%) | 1,619.00 (61.22%) |  |
| missing | 12.00 (0.03%) | 8.00 (0.03%) | 4.00 (0.03%) |  |
| Poverty Ratio |  |  |  | 0.6 |
| ＜1.3 | 3,963.00 (20.22%) | 3,094.00 (20.31%) | 869.00 (19.91%) |  |
| ≥1.3,＜3.5 | 4,742.00 (34.09%) | 3,657.00 (33.76%) | 1,085.00 (35.27%) |  |
| ≥3.5 | 3,711.00 (39.02%) | 2,885.00 (39.29%) | 826.00 (38.02%) |  |
| missing | 1,220.00 (6.67%) | 941.00 (6.63%) | 279.00 (6.80%) |  |
| CKD |  |  |  | <0.001 |
| No | 12,947.00 (96.37%) | 10,208.00 (97.48%) | 2,739.00 (92.35%) |  |
| Yes | 689.00 (3.63%) | 369.00 (2.52%) | 320.00 (7.65%) |  |
| Diabetes |  |  |  | <0.001 |
| No | 10,982.00 (85.94%) | 8,783.00 (87.76%) | 2,199.00 (79.39%) |  |
| Yes | 2,654.00 (14.06%) | 1,794.00 (12.24%) | 860.00 (20.61%) |  |
| Hypertension |  |  |  | <0.001 |
| No | 11,277.00 (86.60%) | 8,920.00 (88.33%) | 2,357.00 (80.37%) |  |
| Yes | 2,359.00 (13.40%) | 1,657.00 (11.67%) | 702.00 (19.63%) |  |
| Hyperlipidemia |  |  |  | <0.001 |
| No | 5,022.00 (37.66%) | 4,227.00 (41.11%) | 795.00 (25.22%) |  |
| Yes | 8,613.00 (62.34%) | 6,350.00 (58.89%) | 2,263.00 (74.78%) |  |
| missing | 1.00 (0.00%) | 0.00 (0.00%) | 1.00 (0.01%) |  |
| CVD |  |  |  | <0.001 |
| No | 12,086.00 (90.86%) | 9,589.00 (92.34%) | 2,497.00 (85.52%) |  |
| Yes | 1,549.00 (9.14%) | 987.00 (7.66%) | 562.00 (14.48%) |  |
| missing | 1.00 (0.00%) | 1.00 (0.00%) | 0.00 (0.00%) |  |

1 Median (IQR); n (unweighted) (%); N (weighted) (%).

2 Wilcoxon rank-sum test for complex survey samples; chi-squared test with Rao & Scott's second-order correction.

Table S4 The relationship between OBS and uricemia through unilinear and unilogistic regression analyses

|  | Serum uric acid level | |  | Hyperuricemia | |
| --- | --- | --- | --- | --- | --- |
| Characteristic | β (95% CI) | p-value |  | OR (95% CI) | p-value |
| continuous Total OBS | -0.02(-0.02, -0.02) | <0.001 |  | 0.97(0.96, 0.98) | <0.001 |
| continuous Dietary OBS | -0.01(-0.02, -0.01) | <0.001 |  | 0.98(0.97, 0.99) | <0.001 |
| continuous Lifestyle OBS | -0.22(-0.24, -0.19) | <0.001 |  | 0.75(0.72, 0.78) | <0.001 |
| Age(year) |  |  |  |  |  |
| 20-39 | ref. |  |  | ref. |  |
| 40-59 | 0.10(0.02, 0.19) | 0.017 |  | 1.15(0.98, 1.35) | 0.094 |
| 60-79 | 0.36(0.28, 0.45) | <0.001 |  | 1.78(1.53, 2.08) | <0.001 |
| 80+ | 0.49(0.38, 0.60) | <0.001 |  | 2.50(2.05, 3.03) | <0.001 |
| Gender |  |  |  |  |  |
| Female | ref. |  |  | ref. |  |
| Male | 1.20(1.20, 1.30) | <0.001 |  | 0.94(0.84, 1.05) | 0.3 |
| Race |  |  |  |  |  |
| Hispanic | ref. |  |  | ref. |  |
| Non-Hispanic white | 0.21(0.13, 0.29) | <0.001 |  | 1.50(1.31, 1.72) | <0.001 |
| Non-Hispanic black | 0.22(0.13, 0.30) | <0.001 |  | 1.70(1.47, 1.98) | <0.001 |
| other races | 0.24(0.15, 0.33) | <0.001 |  | 1.43(1.19, 1.71) | <0.001 |
| Education |  |  |  |  |  |
| Below high school | ref. |  |  | ref. |  |
| High school | 0.04(-0.05, 0.13) | 0.4 |  | 1.11(0.96, 1.29) | 0.14 |
| Above high school | -0.03(-0.09, 0.04) | 0.4 |  | 1.05(0.92, 1.20) | 0.5 |
| Poverty Ratio |  |  |  |  |  |
| (0,1.3) | ref. |  |  | ref. |  |
| [1.3, 3.5) | 0.11(0.04, 0.19) | 0.005 |  | 1.07(0.92, 1.24) | 0.4 |
| ≥3.5 | 0.11(0.03, 0.20) | 0.01 |  | 0.99(0.86, 1.14) | 0.9 |
| CKD |  |  |  |  |  |
| No | ref. |  |  | ref. |  |
| Yes | 0.84(0.67, 0.99) | <0.001 |  | 3.20(2.60, 3.96) | <0.001 |
| Diabetes |  |  |  |  |  |
| No | ref. |  |  | ref. |  |
| Yes | 0.43(0.33, 0.52) | <0.001 |  | 1.86(1.62, 2.14) | <0.001 |
| Hypertension |  |  |  |  |  |
| No | ref. |  |  | ref. |  |
| Yes | 0.39(0.30, 0.49) | <0.001 |  | 1.85(1.61, 2.12) | <0.001 |
| Hyperlipidemia |  |  |  |  |  |
| No | ref. |  |  | ref. |  |
| Yes | 0.38(0.32, 0.45) | <0.001 |  | 2.07(1.79, 2.40) | <0.001 |
| CVD |  |  |  |  |  |
| No | ref. |  |  | ref. |  |
| Yes | 0.54(0.41, 0.67) | <0.001 |  | 2.04(1.77, 2.35) | <0.001 |
| Energy intake quartiles |  |  |  |  |  |
| Q1 (0,1522.50) | ref. |  |  | ref. |  |
| Q2 [1522.50,1971.00) | 0.07(-0.01, 0.16) | 0.084 |  | 0.84(0.73, 0.97) | 0.015 |
| Q3 [1971.00, 2507.50) | 0.22(0.13, 0.31) | <0.001 |  | 0.79(0.67, 0.92) | 0.003 |
| Q4 ≥ 2507.50 | 0.46(0.38, 0.54) | <0.001 |  | 0.71(0.61, 0.83) | <0.001 |
| Caffeine intake quartiles |  |  |  |  |  |
| Q1 (0, 39.50) | ref. |  |  | ref. |  |
| Q2 [39.50, 121.50) | 0.18(0.11, 0.26) | <0.001 |  | 1.26(1.09, 1.45) | 0.002 |
| Q3 [121.50, 235.50) | 0.17(0.08, 0.25) | <0.001 |  | 1.11(0.94, 1.31) | 0.2 |
| Q4 ≥ 235.50 | 0.30(0.21, 0.40) | <0.001 |  | 1.20(1.01, 1.43) | 0.04 |
| Protein intake quartiles |  |  |  |  |  |
| Q1 (0, 57.84) | ref. |  |  | ref. |  |
| Q2 [57.84,76.51) | 0.13(0.04, 0.22) | 0.005 |  | 0.90(0.77, 1.05) | 0.2 |
| Q3 [76.51,98.67) | 0.32(0.24, 0.40) | <0.001 |  | 0.90(0.78, 1.05) | 0.2 |
| Q4 ≥ 98.67 | 0.52(0.42, 0.62) | <0.001 |  | 0.79(0.67, 0.94) | 0.008 |
| Sugar intake quartiles |  |  |  |  |  |
| Q1 (0, 63.60) | ref. |  |  | ref. |  |
| Q2 [63.60,96.11) | -0.15(-0.26, -0.04) | 0.007 |  | 0.74(0.63, 0.87) | <0.001 |
| Q3 [96.11, 137.28) | -0.14(-0.25, -0.03) | 0.011 |  | 0.65(0.54, 0.79) | <0.001 |
| Q4 ≥ 137.28 | 0.03(-0.09, 0.14) | 0.6 |  | 0.66(0.55, 0.78) | <0.001 |
| Lifestyle OBS quartiles |  |  |  |  |  |
| Q1 [0, 3) | ref. |  |  | ref. |  |
| Q2 [3, 4) | -0.31(-0.41, -0.21) | <0.001 |  | 0.66(0.57, 0.76) | <0.001 |
| Q3 [4, 6) | -0.62(-0.71, -0.54) | <0.001 |  | 0.43(0.37, 0.51) | <0.001 |
| Q4 ≥ 6 | -1.00(-1.20, -0.89) | <0.001 |  | 0.24(0.18, 0.31) | <0.001 |
| Dietary OBS quartiles |  |  |  |  |  |
| Q1 [0, 10) | ref. |  |  | ref. |  |
| Q2 [10, 16) | -0.06(-0.15, 0.02) | 0.12 |  | 0.91(0.79, 1.05) | 0.2 |
| Q3 [16, 22) | -0.07(-0.16, 0.01) | 0.086 |  | 0.89(0.77, 1.03) | 0.12 |
| Q4 ≥ 22 | -0.25(-0.35, -0.16) | <0.001 |  | 0.66(0.55, 0.81) | <0.001 |
| Total OBS quartiles |  |  |  |  |  |
| Q1 [0, 14) | ref. |  |  | ref. |  |
| Q2 [14, 20) | -0.10(-0.19, -0.01) | 0.028 |  | 0.86(0.75, 0.98) | 0.03 |
| Q3 [20, 26) | -0.16(-0.25, -0.07) | <0.001 |  | 0.81(0.69, 0.94) | 0.006 |
| Q4 ≥ 26 | -0.39(-0.49, -0.30) | <0.001 |  | 0.56(0.46, 0.68) | <0.001 |
| ^1^CI = Confidence Interval, OR = Odds Ratio | | | | | |

OR, odds ratio; CI, confidence intervals; OBS, oxidative balance score.

Table S5 Sensitivity analyses of uricemia and hyperuricemia outcomes by sequential elimination of individual OBS components.

| OBS component  excluded | uricemia | | hyperuricemia | | | |
| --- | --- | --- | --- | --- | --- | --- |
|  | β (95% CI) | p-value | | OR (95% CI) | p-value | |
| Physical activity | -0.02(-0.03, -0.02) | <0.001 | | 0.97(0.96, 0.98) | | <0.001 |
| cotinine | -0.02(-0.03, -0.02) | <0.001 | | 0.97(0.96, 0.98) | | <0.001 |
| Alcohol | -0.02(-0.02, -0.01) | <0.001 | | 0.97(0.96, 0.98) | | <0.001 |
| Body mass index | -0.01(-0.02, -0.01) | <0.001 | | 0.98(0.97, 0.99) | | <0.001 |
| Dietary fiber | -0.02(-0.03, -0.02) | <0.001 | | 0.97(0.96, 0.98) | | <0.001 |
| Carotene | -0.02(-0.03, -0.02) | <0.001 | | 0.97(0.96, 0.98) | | <0.001 |
| Vitamin B6 | -0.02(-0.03, -0.02) | <0.001 | | 0.96(0.95, 0.98) | | <0.001 |
| Vitamin B12 | -0.02(-0.03, -0.02) | <0.001 | | 0.96(0.95, 0.98) | | <0.001 |
| Riboflavin | -0.02(-0.03, -0.02) | <0.001 | | 0.97(0.95, 0.98) | | <0.001 |
| Total Folate | -0.02(-0.03, -0.02) | <0.001 | | 0.97(0.96, 0.98) | | <0.001 |
| Niacin | -0.02(-0.03, -0.02) | <0.001 | | 0.96(0.95, 0.97) | | <0.001 |
| Vitamin C | -0.02(-0.03, -0.02) | <0.001 | | 0.97(0.95, 0.98) | | <0.001 |
| Vitamin E | -0.02(-0.03, -0.02) | <0.001 | | 0.97(0.96, 0.98) | | <0.001 |
| Calcium | -0.02(-0.03, -0.02) | <0.001 | | 0.97(0.96, 0.98) | | <0.001 |
| Magnesium | -0.02(-0.03, -0.02) | <0.001 | | 0.97(0.95, 0.98) | | <0.001 |
| Zinc | -0.02(-0.03, -0.02) | <0.001 | | 0.96(0.95, 0.98) | | <0.001 |
| Copper | -0.02(-0.03, -0.02) | <0.001 | | 0.96(0.95, 0.98) | | <0.001 |
| Selenium | -0.02(-0.03, -0.02) | <0.001 | | 0.97(0.95, 0.98) | | <0.001 |
| Iron | -0.02(-0.03, -0.02) | <0.001 | | 0.97(0.96, 0.98) | | <0.001 |
| Total fat | -0.02(-0.02, -0.02) | <0.001 | | 0.97(0.96, 0.98) | | <0.001 |

CI = Confidence Interval, OR = Odds Ratio
